# Immortalisation of primary human alveolar epithelial lung cells using a non-viral vector to study respiratory bioreactivity in vitro

**DOI:** 10.1038/s41598-020-77191-y

**Published:** 2020-11-24

**Authors:** Alberto Katsumiti, Pakatip Ruenraroengsak, Miren P. Cajaraville, Andrew J. Thorley, Teresa D. Tetley

**Affiliations:** 1 CBET Research Group, Department of Zoology and Animal Cell Biology, Faculty of Science and Technology and Research Centre for Experimental Marine Biology and Biotechnology PiE, University of the Basque Country UPV/EHU, Plentzia, Basque Country Spain; 2 National Heart and Lung Institute, Imperial College London, London, SW7 2AZ UK; 3 Department of Materials and London Centre for Nanotechnology, Imperial College London, London, SW7 2AZ UK; 4 Department of Pharmacy, Faculty of Pharmacy, Mahidol University, 447 Sri-Ayuthaya Road, Rajathevi, Bangkok, 10400 Thailand

**Keywords:** Biological techniques, Cell biology, Stem cells, Molecular medicine

## Abstract

To overcome the scarcity of primary human alveolar epithelial cells for lung research, and the limitations of current cell lines to recapitulate the phenotype, functional and molecular characteristics of the healthy human alveolar epithelium, we have developed a new method to immortalise primary human alveolar epithelial lung cells using a non-viral vector to transfect the telomerase catalytic subunit (hTERT) and the simian virus 40 large-tumour antigen (SV40). Twelve strains of immortalised cells (ICs) were generated and characterised using molecular, immunochemical and morphological techniques. Cell proliferation and sensitivity to polystyrene nanoparticles (PS) were evaluated. ICs expressed caveolin-1, podoplanin and receptor for advanced glycation end-products (RAGE), and most cells were negative for alkaline phosphatase staining, indicating characteristics of AT1-like cells. However, most strains also contained some cells that expressed pro-surfactant protein C, classically described to be expressed only by AT2 cells. Thus, the ICs mimic the cellular heterogeneity in the human alveolar epithelium. These ICs can be passaged, replicate rapidly and remain confluent beyond 15 days. ICs showed differential sensitivity to positive and negatively charged PS nanoparticles, illustrating their potential value as an in vitro model to study respiratory bioreactivity. These novel ICs offer a unique resource to study human alveolar epithelial biology.

## Introduction

The respiratory alveolar epithelium is composed of alveolar type 1 (AT1) and type 2 (AT2) cells. AT1 are flattened cells (~ 0.2 µm deep, 40–80 µm across) which cover 95% of the alveolar surface, providing a large surface area for gas exchange^1^. They are involved in protein transport and translocation via transcytosis^2^ and regulate fluid fluxes and ion transport^1^, and emerging evidence suggests they are involved in alveolar maturation and modulation of immune functions.

AT2 cells are cuboidal secretory cells which synthesise and release pulmonary surfactant, essential for the maintenance of alveolar epithelial homeostasis, maintaining reduced surface tension, preventing alveolar collapse during ventilation^3^. It is stored within lamellar bodies and consists of ~ 90% phospholipids and 10% protein, including surfactant proteins (SP) A, B, C and D^4,5^; SPs are essential for normal surfactant function, as well as having a crucial role in immune defense. AT2 cells have a key role in maintaining alveolar epithelial structural integrity, acting as alveolar progenitors, proliferating and differentiating into AT1 cells repopulating injured epithelium^3,6,7^.

Unlike many other primary cells, primary human pulmonary alveolar epithelial cells exhibit very limited proliferation in vitro and therefore cannot be passaged and expanded for extensive studies. Consequently, they need to be regularly isolated from lung tissue for each experimental set, which limits the scope of the research in this area. Therefore, there is considerable interest in the generation of well characterised alveolar epithelial cell lines derived from primary human lung cells.

Currently, there are few alveolar epithelial cell lines derived from human lung tissue. The A549 cell line, established in 1972 by Giard et al.^8^ is the most frequently used human alveolar cell model for biopharmaceutical research. It was derived from a human pulmonary adenocarcinoma and originally exhibited some morphological and biochemical features of AT2 cells, containing lamellar bodies and SPs^9,10^. The NCI-H441 cell line was isolated in 1982^11^ from the pericardial fluid of a patient with papillary pulmonary adenocarcinoma and showed characteristics of both AT2 and Club cell-like bronchiolar epithelial cells^12^. More recently, Kemp et al.^13^ immortalised AT2 cells from normal tissue through viral transfection of the catalytic subunit of telomerase (hTERT) and the temperature-sensitive mutant of simian virus 40 large-tumour antigen (SV40). This cell exhibited AT1-like cell phenotype and was termed as a transformed type 1 (TT1) cell line^13,14^.

Most current protocols to immortalise primary cells are based on viral vectors^15^. These are widely applied in gene therapy, however their marked immunogenicity causes induction of proinflammatory responses and degeneration of transfected cells^15,16^. Furthermore, there is a potential risk of insertional mutagenesis, raising important concerns on their safe use in humans, effects which likely apply to isolated primary cell preparations^17^. Non-viral methods based on cationic liposomes and cationic nanoparticles, e.g. lipofectamine, have been increasingly used in drug and gene delivery due to their low toxicity, lack of activation of the immune system and high capacity to deliver both hydrophilic and hydrophobic active compounds^15,17,18^, thus providing a suitable tool for immortalisation of isolated primary human cells.

In the present work, we aimed to establish a new method to immortalise primary human AT2 cells based on non-viral transfections with lipofectamine™. Subsequently, newly immortalised cells (ICs) were characterised based on molecular, immunochemical and morphological techniques to identify cell phenotype. Their phenotypes were also compared with our typical viral vector immortalised cells and primary human AT2 cells. ICs’ proliferation capacity in vitro was also assessed. Finally, ICs were exposed to nano-sized surface-modified polystyrene (PS) particles in order to evaluate their suitability to be used as model for in vitro bioreactivity and toxicity testing.

## Results

### Characterisation of non-viral transfection vector

According to dynamic light scattering (DLS) analysis, lipofectamine (LF), cationic lipidic particles, showed hydrodynamic size varying from 89.58 to 115.6 nm with polydispersity index (PDI) between 0.422 and 0.503 and zeta potential values ranging from + 68.4 to + 81.6 mV in the different diluents (Table 1; Supplementary Fig. S1a–c online). Once mixed with plasmid DNA, particles showed a polydispersed distribution with PDI ranging from 0.261 to 0.455 (Supplementary Fig. S1d–f online), with an increased average diameter (177 to 198.7 nm) and decreased zeta potential values (+ 0.0966 to + 23 mV) compared to LF particles alone (Table 1), confirming the formation of DNA-lipid complexes (lipoplex). Among the three LF to DNA dilution ratios (1:1, 1:2 and 1:4, Supplementary Fig. S1d–f online), ratios 1:4 followed by 1:2 liposomes to nucleic acid were best for formation of nanomeric complexes (Supplementary Fig. S1e,f online). Based on surface charge, 1:1 and 1:2 liposomes to nucleic acid ratios showed high positive zeta potential values (Table 1), whereas 1:4 ratio exhibited an almost neutral surface charge (Table 1). This may be explained by the change in zeta potential values where the ratio (LF:DNA) was 1:1 and 1:2 the complex (lipoplex) between cationic lipidic-molecule (LF) and DNA due to electrostatic interaction between the amine functional group of LF molecule and anionic phospholipid group of DNA. When the DNA was condensed the zeta potential exhibited a positive surface charge density (+ 22 (1:1 ratio) and + 23 (1:2 ratio) mV) as seen in Table 1. The increase in amount of DNA beyond 1:2 ratio would need more LF to interact and condense DNA as the zeta potential was reduced at 1.4 ratio, thus 1:2 was deemed the best liposome to nucleic acid ratio for transfections.

**Table 1:** Hydrodynamic size (diameter) and zeta potential of different mixing ratios of dilution of LF and LF to DNA in Opti-MEM medium.

Samples	Average diameter (nm ± SD)	Polydispersity index (PDI)	Zeta potential (mV ± SD)
1:33 (LF:DW)	145.2 ± 3.717	0.264 ± 0.029	+ 45.6 ± 1.37
1:16 (LF:DW)	102.9 ± 1.888	0.432 ± 0.016	+ 72.1 ± 2.13
1:8 (LF:DW)	68.77 ± 2.637	0.374 ± 0.060	+ 84.9 ± 5.57
1:33 (LF:OptiMEM)	97.84 ± 5.293	0.503 ± 0.042	+ 68.4 ± 3.48
1:16 (LF:OptiMEM)	115.6 ± 2.608	0.448 ± 0.071	+ 72.7 ± 8.72
1:8 (LF:OptiMEM)	89.58 ± 5.712	0.422 ± 0.007	+ 81.6 ± 6.20
1:1 (LF:DNA)	198.7 ± 3.035	0.261 ± 0.004	+ 22.2 ± 0.0577
1:2 (LF:DNA)	177.0 ± 3.439	0.292 ± 0.013	+ 23.0 ± 0.265
1:4 (LF:DNA)	180.0 ± 5.567	0.455 ± 0.015	+ 0.0966 ± 0.192

Data are addressed as mean ± SD, n = 3.

**Figure 1:**
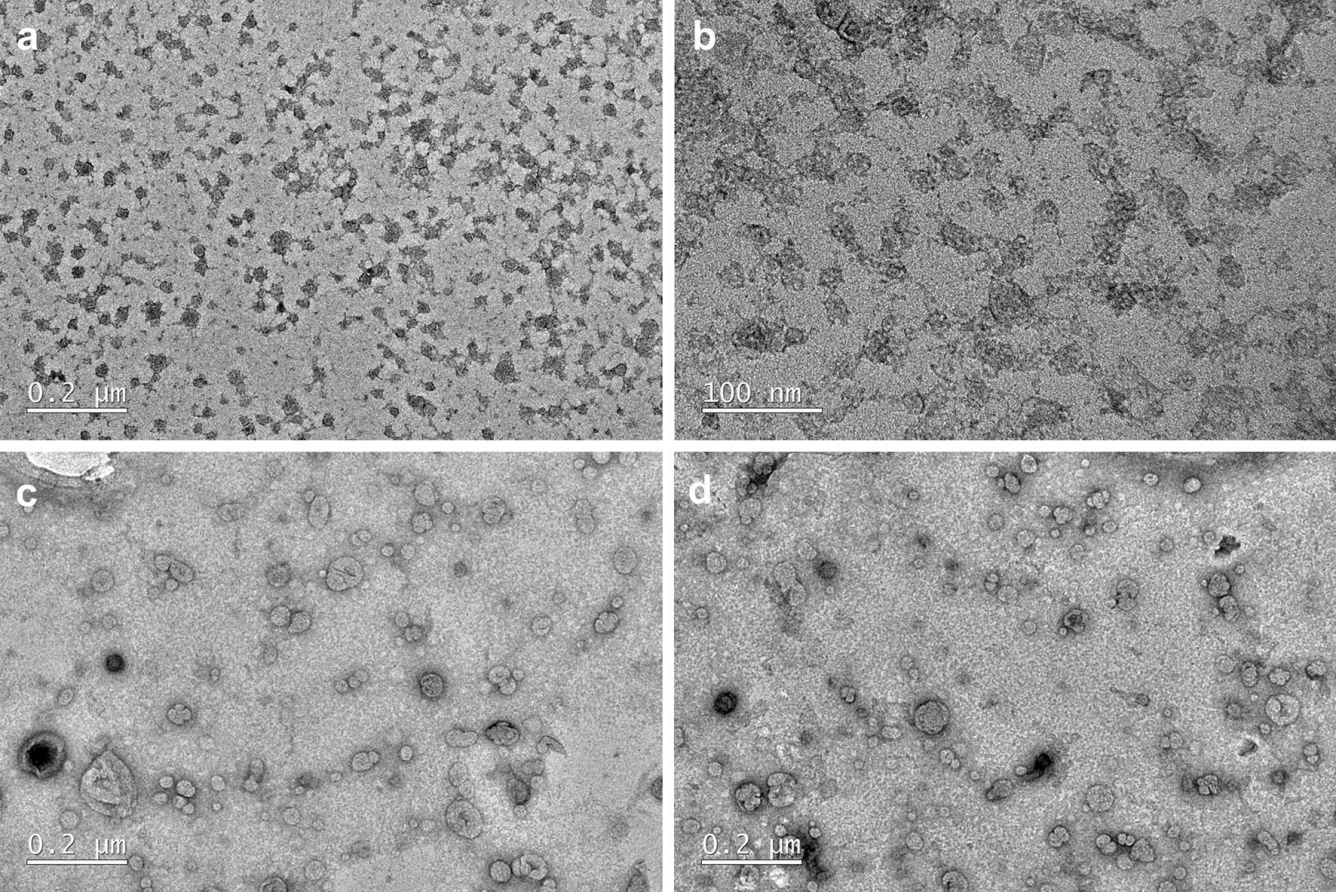
TEM images of LF particles alone (**a**,**b**) and in nanomeric complex with DNA (**c**,**d**).

Based on the TEM analysis, LF particles exhibited an irregular shape of less than 50 nm (35.01 ± 9.77) (Fig. 1a,b). Once mixed with DNA, nanomeric complexes appeared as rounded particles, up to 100 nm in size, showing electron-lucid core DNA, wrapped in electron-dense lipid (Fig. 1c,d).

### Non-viral transfections and characterisation of ICs

Transfections successfully occurred in cultures with 30%, 50% and 70% of confluency, using all three LF:DNA ratios (1:1, 1:2 and 1:4). Best results *(*higher transfection efficiency*)* were obtained in cultures with *30%* of confluency using a LF:DNA ratio of 1:2. Twelve IC strains were obtained from primary AT2 cells isolated from the lung tissue of one donor and characterised separately as human alveolar epithelial lung cell line, HAEL-1 to HAEL-12.

### Gene transcription levels

**Figure 2:**
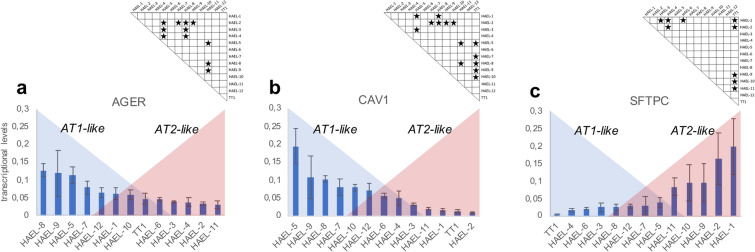
Relative transcript levels of RAGE (**a**), caveolin-1 (**b**) and SP-C (**c**) in ICs and TT1 cell line. Values are given as means ± SD. Stars indicate significant differences (p < 0.05) among groups according to the Kruskal–Wallis followed by the Dunn’s post hoc test. n = 6 replicates per sample.

Gene expression of the phenotypic markers, RAGE (AGER), caveolin-1 (CAV1) and SP-C (SFTPC) was assessed in ICs and TT1 cell lines (Fig. 2). AGER transcription levels varied among ICs, the highest being ~ 5 times that of the lowest, but all ICs expressed AGER, and most were not significantly different to that of TT1 (Fig. 2a). HAEL-5, HAEL-8 and HAEL-9 ICs showed the highest AGER transcriptional levels, markedly more than TT1 cells, whereas HAEL-11 ICs exhibited the lowest expression (Fig. 2a). CAV1 transcriptional levels differed between ICs and TT1 cells (Fig. 2b). HAEL-5, HAEL-7, HAEL-8, HAEL-9 and HAEL-10 ICs showed statistically higher CAV1 transcriptional levels than TT1 cells (p < 0.05, n = 6) Fig. 2b). TT1 together with HAEL-1, HAEL-2 and HAEL-11 ICs showed the lowest CAV1 transcriptional levels (Fig. 2b). SFTPC was expressed in all ICs but not in TT1 cells (Fig. 2c). Transcriptional levels of SFTPC in HAEL-1, HAEL-2, HAEL-9, HAEL-10 and HAEL-11 ICs were statistically higher compared to TT1 cells (Fig. 2c). The remaining ICs expressed very low levels of SFTPC.

### Western blotting

**Figure 3:**
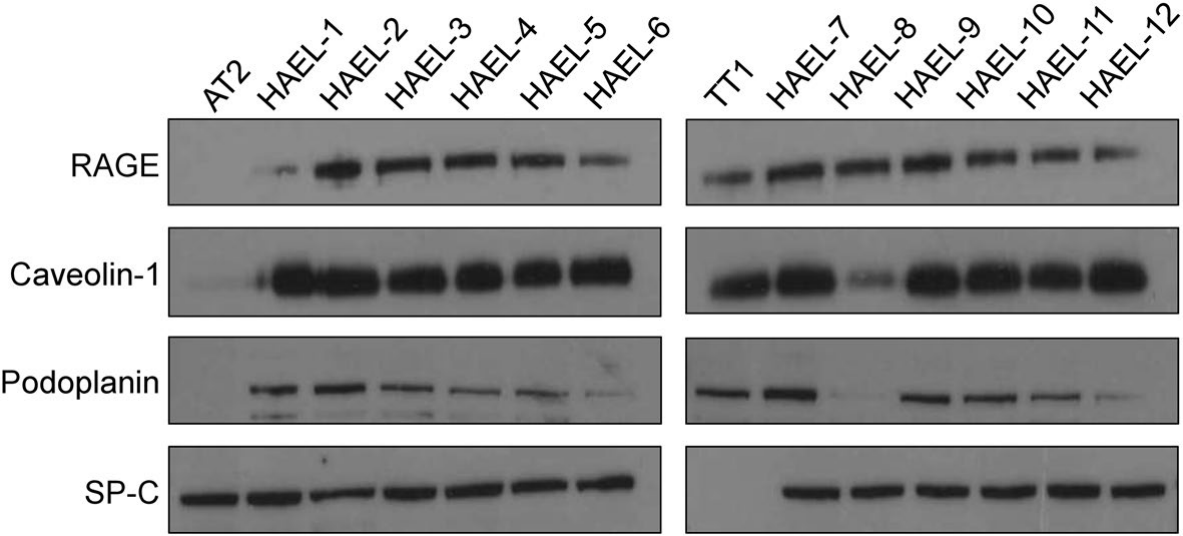
Immunoblotting analysis of RAGE, caveolin-1, podoplanin and SP-C in ICs, TT1 cell line and primary AT2 cells. Loading protein concentrations were adjusted to 50 μg per sample.

Immunoblotting of ICs, TT1 cell line and primary AT2 cells for RAGE, caveolin-1, podoplanin and SP-C is shown in Fig. 3 and in Supplementary Fig. S2 online. RAGE and caveolin-1 were strongly expressed in most ICs and in TT1 cells except for HAEL-1 (for RAGE) and HAEL-8 (for caveolin-1), which was lower (Fig. 3; Supplementary Fig. S2 online). Podoplanin was expressed in eleven of the twelve ICs and also in TT1 cells; expression varied between strains (Fig. 3; Supplementary Fig. S2 online). As expected, RAGE, caveolin-1 and podoplanin were not expressed in primary AT2 cells (Fig. 3; Supplementary Fig. S2 online). SP-C was found in all ICs and in AT2 cells but not in TT1 cells (Fig. 3; Supplementary Fig. S2 online).

### Immunocytochemistry

**Figure 4:**
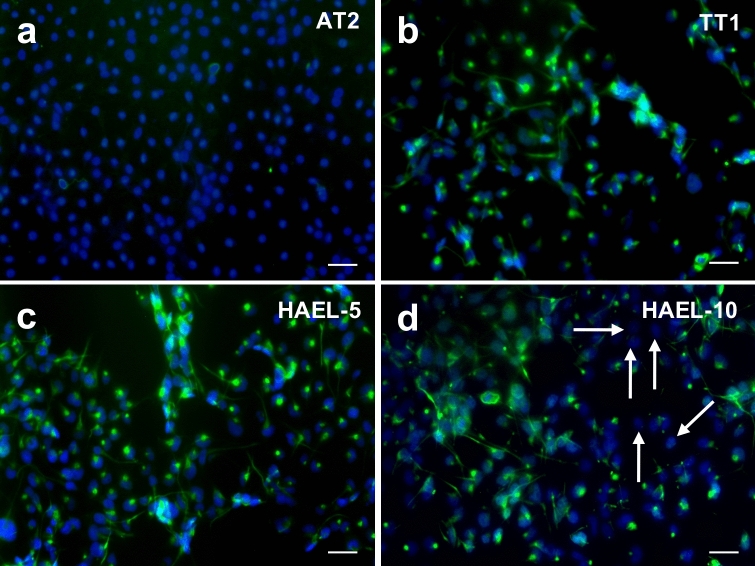
Immunofluorescent labelling of podoplanin (green) in AT2 cells (**a**), TT1 cell line (**b**) and ICs (**c**,**d**). Podoplanin is present in most of the cells but not in all of them (white arrows). Cell nuclei are stained blue. Bars = 50 µm.

*Immunocytochemistry* of podoplanin in ICs, TT1 cell line and primary AT2 cells (Fig. 4) demonstrated podoplanin in all IC strains and TT1 cells but not in AT2 cells (Fig. 4). Within ICs, podoplanin was present in most of the cells but not all of them (white arrows, Fig. 4d); 6.33 ± 2.38% were negative for podoplanin.

### Alkaline phosphatase

**Figure 5:**
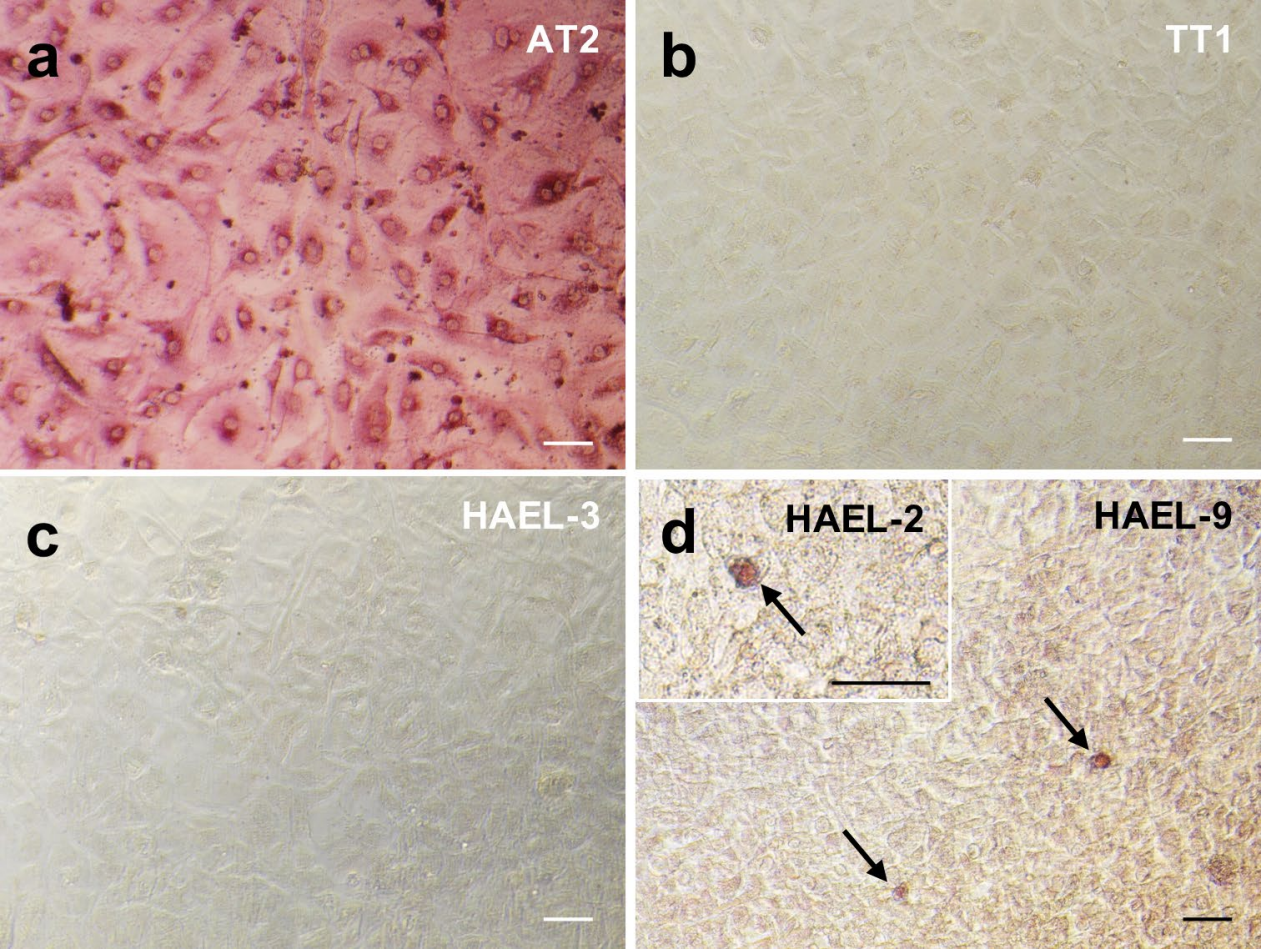
Alkaline phosphatase staining showing AP-positive AT2 cells (**a**) and negative TT1 cells (**b**). Some ICs were AP-negative (**c**) whereas others were mostly AP-negative showing scattered AP-positive cells (arrows) (**d**). Bars = 50 µm.

Alkaline phosphatase was assessed in ICs, TT1 cell line and primary AT2 cells (Fig. 5). AT2 cells exhibited strong pink AP-positive staining (Fig. 5a), whereas TT1 cells were AP-negative (Fig. 5b). IC strains HAEL-3, HAEL-4 and HAEL-12 were AP-negative (e.g. HAEL-3, Fig. 5c). HAEL-1, HAEL-2, HAEL-5, HAEL-6, HAEL-7, HAEL-8, HAEL-9, HAEL-10 and HAEL-11 strains were mostly AP-negative but contained scattered AP-positive cells (e.g. HAEL-2 and 9, white arrows, Fig. 5d).

### TEM analysis

**Figure 6:**
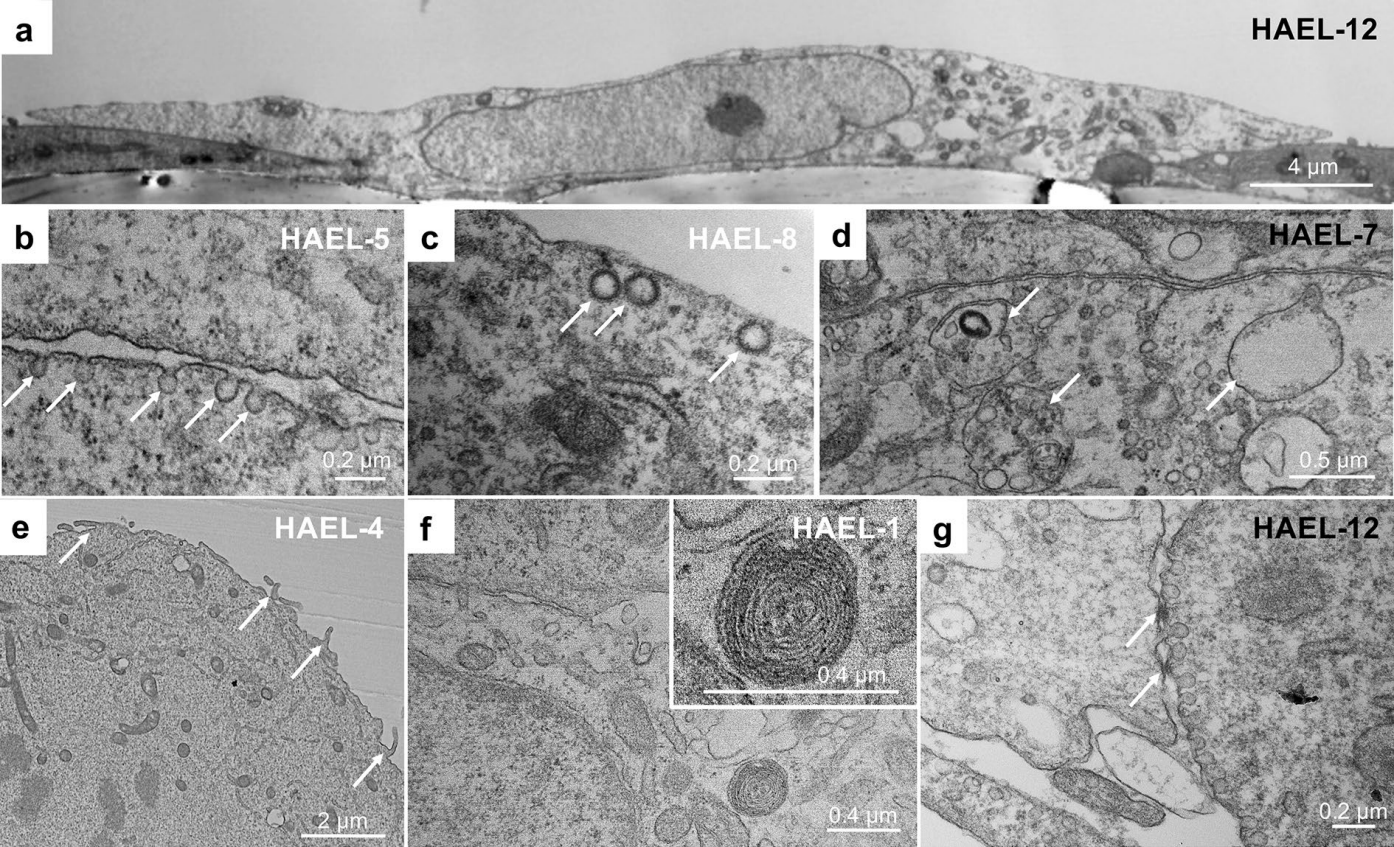
TEM images of ICs showing a flattened thin morphology (**a**), membrane invaginations (caveolae, arrows) (**b**), clathrin-coated vesicles (arrows) (**c**) and numerous endosomal vesicles (arrows) (**d**). Some cells showed a different morphology with the presence of apical microvilli (arrows) (**e**), cytoplasmic inclusions resembling lamellar bodies (**f**) and tight junctions (**g**).

At low magnification, most of the ICs showed a flattened squamous morphology (Fig. 6a). Membrane invaginations such as flask-shaped caveolae (Fig. 6b) and clathrin coated pits (Fig. 6c) were found at the apical and basal plasma membrane of ICs, with numerous endosomal vesicles within the cytoplasm (Fig. 6d). These are all features of AT1 cells. In some strains (HAEL-1, HAEL-2, HAEL-4, HAEL-11 and HAEL-12), a few cells showed apical microvilli (Fig. 6e). Lamellar bodies (Fig. 6f), features of AT2 cells^13^, were found in HAEL-1, HAEL-2, HAEL-4, HAEL-9, HAEL-10, HAEL-11 and HAEL-12. Strong tight junctions (Fig. 6g) were observed in HAEL-1, HAEL-2, HAEL-4, HAEL-5, HAEL-11 and HAEL-12 strains, as shown by us previously in AT2 preparations in vitro^19^.

### Cell proliferation

**Figure 7:**
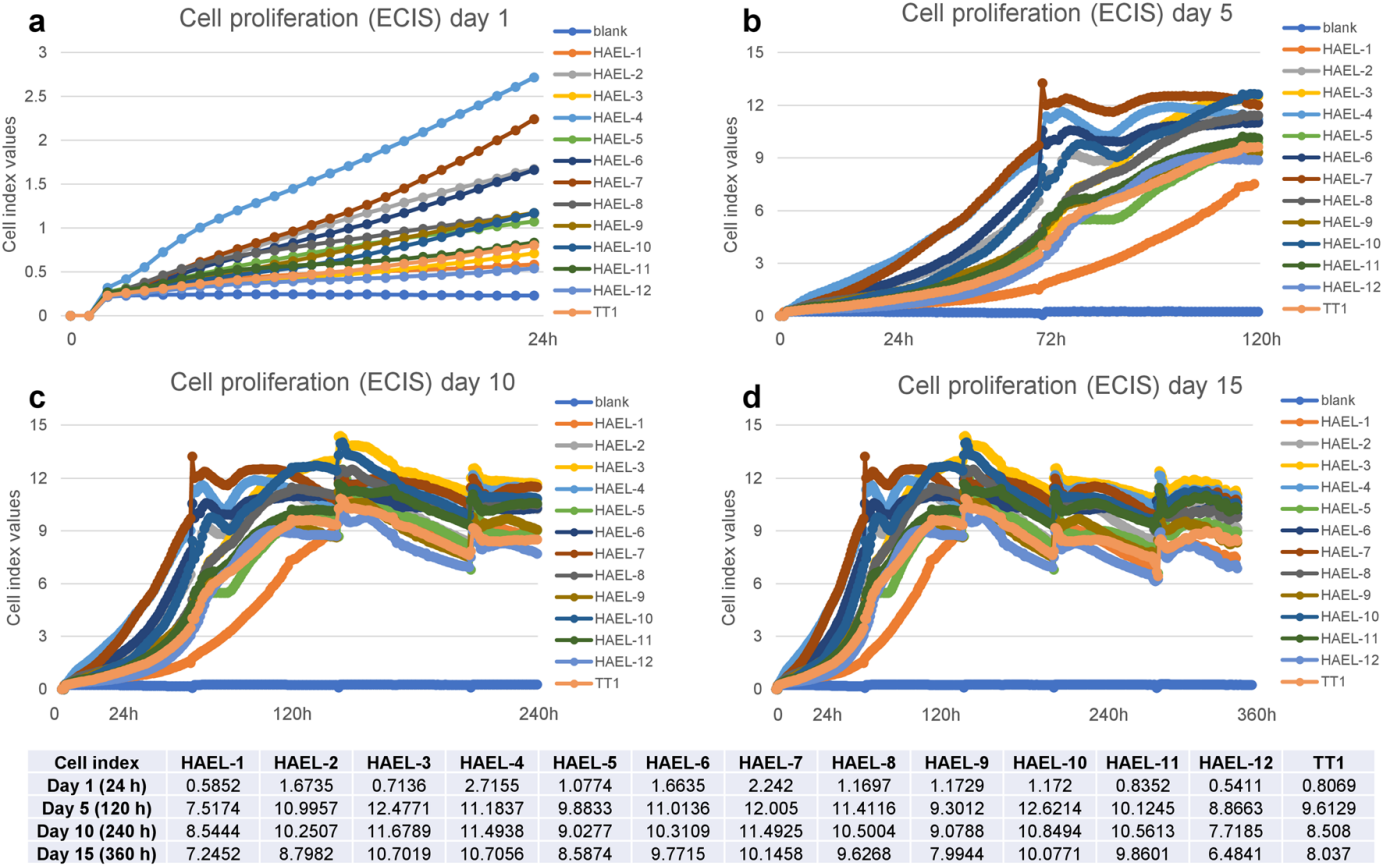
Cell proliferation given as cell index of ICs and TT1 cells after 1 (**a**), 5 (**b**), 10 (**c**) and 15 (**d**) days (media changed every 3 days).

Proliferation of ICs and TT1 cells was measured over fifteen days. Cell culture medium was renewed every three days. The cells replicated rapidly and then remained confluent (Fig. 7). Cells proliferated at different rates, especially during the first 24 h (Fig. 7a). Generally, HAEL-2, HAEL-4, HAEL-5, HAEL-6, HAEL-7, HAEL-8, HAEL-10 and HAEL-11 strains propagated faster than TT1 cells; HAEL-3 and HAEL-9 propagation varied over time, while HAEL-1 and HAEL-12 strains showed the lowest proliferation rate (Fig. 7). After five days (120 h), cell proliferation increased continuously, with a slight oscillation at day three (72 h), associated with a medium change (Fig. 7b). This was also observed during other medium changes during the experiment (Fig. 7c,d) and could be related to some cell loss from the monolayers during washing, followed by recovery of monolayer integrity. Cell proliferation plateaued around day five (120 h), having achieved a confluent monolayer (Fig. 7c). After reaching confluence, cell proliferation fluctuated with time (Fig. 7d), however monolayers maintained their integrity due to the regular medium change every three days.

### In vitro toxicity testing (MTT assay)

**Figure 8:**
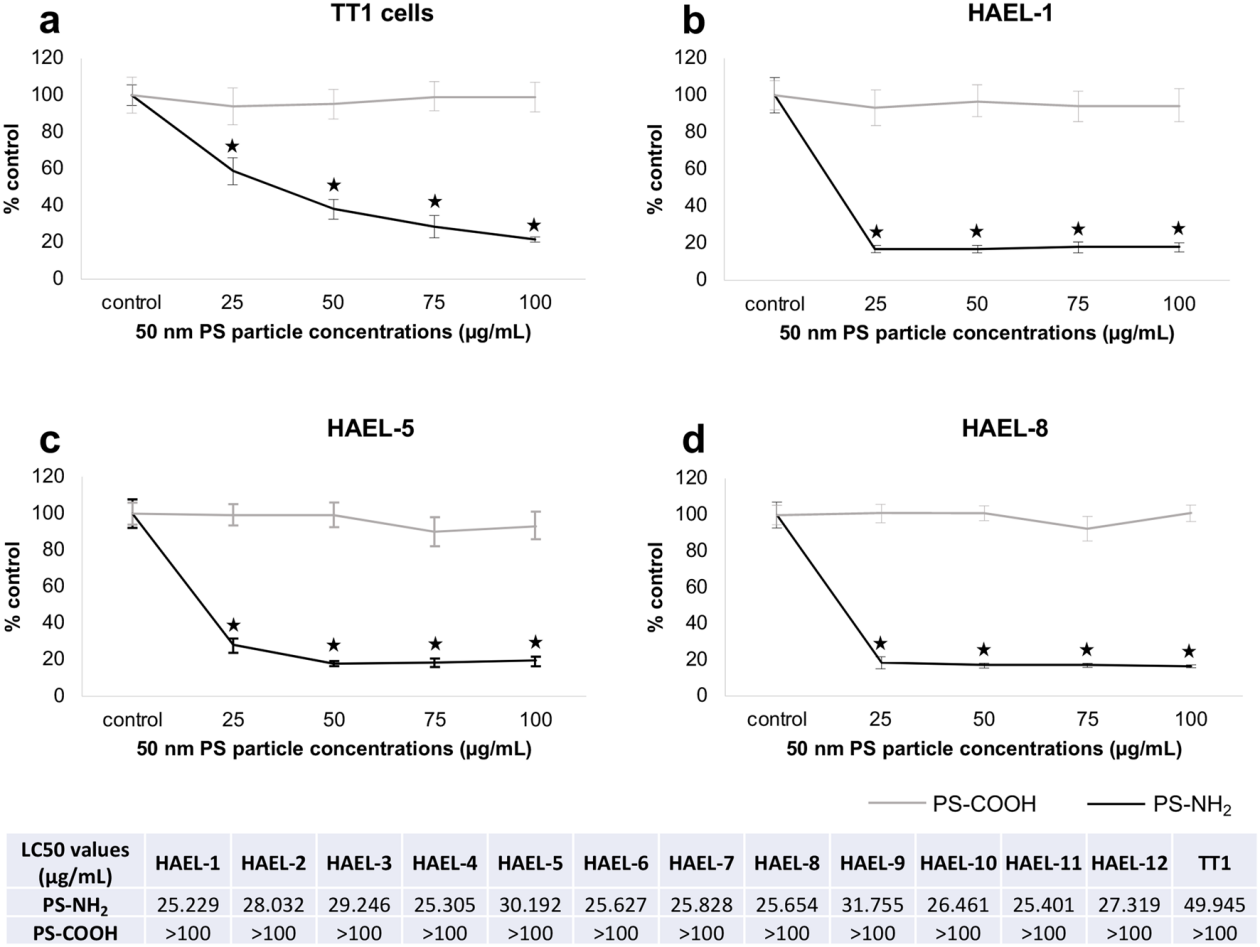
Cell viability (MTT assay) of TT1 cells (**a**) and ICs (**b**–**d**) exposed to 50 nm PS-NH_2_ and PS-COOH particles for 24 h. Data are given as percentage with respect to controls (means ± SD). Stars indicate significant differences (p < 0.05) in treated cells with respect to controls according to the Kruskal–Wallis followed by the Dunn’s post hoc test. n = 6 replicates per treatment. LC50 values were calculated through Probit analysis.

Cytotoxicity of 50 nm PS-NH_2_ and PS-COOH was assessed on ICs and the TT1 cell line (Fig. 8). PS-COOH were not cytotoxic at any concentration tested (Fig. 8). In contrast, PS-NH_2_ were extremely toxic to ICs and TT1 cells at all tested concentrations, reducing cell viability by up to 80% (Fig. 8). All IC strains showed similar sensitivity to PS-NH_2_ at the tested concentrations. Despite this, HAEL-1 showed slightly higher sensitivity to PS- NH_2_ (LC50 = 25.229 µg/mL) compared to other strains such as HAEL-5 (LC50 = 30.192 µg/mL) and HAEL-9 (LC50 = 31.755 µg/mL). Comparing the sensitivity of ICs and TT1 cells, the former were more sensitive than the latter at concentrations below 100 µg/mL (Fig. 8).

## Discussion

We have successfully developed a new technique to immortalise primary human pulmonary alveolar epithelial type 2 cells (AT2) cells using a non-viral vector to transfect hTERT and SV40 into primary cells, generating twelve novel human alveolar epithelial cell lines.

Here, use of cationic liposomes resulted in efficient transfection, which is mainly dependent on their size and surface charge, being incorporated into cells mainly through endocytic mechanisms. There is a general consensus that submicron particles exhibit higher rates of endocytosis than micron-sized particles^17,20^. The TEM images show nanomeric complexes of 100 nm and less, thus at the recommended sub-micron range of size. DLS analysis showed nanomeric complexes with higher hydrodynamic diameters (177.0 to 198.7 nm), likely reflecting adsorption of proteins and other medium components on particle surfaces, but still maintaining the sub-micron size range. Liposome particles were highly positively charged, ranging from + 68.4 to + 81.6 mV. Mixing with plasmid DNA (ratios 1:1, 1:2 and 1:4; lipid:nucleic acid), resulted in decreased zeta potential (+ 0.0966 to + 23 mV). The ratio 1:4 followed by 1:2 showed the best results concerning the formation of nanomeric complexes. The ratios 1:1 and 1:2 showed higher positive zeta potential values compared to 1:4 ratio (almost neutral). According to Murugan et al.^20^, both cationic and neutral particles show higher internalisation efficiency due to attraction to the negatively charged cell membrane. Similarly, highly positively charged particles should show greater rate and extent of internalisation compared to less positive particles, thus representing better vectors for transfection. In our study, although transfections were achieved at all tested liposomes:nucleic acid ratios, better results were obtained using the highest positively charged nanomeric complexes (+ 22.2 and 23.0 mV). Thus, taking both results into account (efficiency of nanomeric complex formation and surface charge) 1:2 liposomes:nucleic acid ratio was chosen as the best ratio for DNA transfections.

Transfections successfully occurred in cells seeded at 30%, 50% and 70% of confluency, using 1:1, 1:2 and 1:4 LF:DNA ratios, however, transfection efficiency was higher in cells seeded at 30% of confluency using 1:2 LF:DNA ratio, thus this was selected as the optimum condition. Immortalised ICs showed mostly alveolar AT1-like characteristics, although AT2-like characteristics were also expressed by ICs. Gene expression and immunoblotting analysis showed that all ICs expressed RAGE and caveolin-1, expressed by TT1 cells but not AT2 cells. TT1 and AT2 cell expression profiles agreed with our previous studies^13^, and with other comparative studies of AT1 and AT2 cells^1,3^. Regarding AGER expression*,* no significant differences were found between ICs and TT1 cells. RAGE is a member of the immunoglobulin superfamily originally identified for its ability to bind advanced glycation end-products, which are formed by glycosidation^21^. RAGE expression enhances adherence of epithelial cells to collagen-coated surfaces and induces cell spreading^22,23^; its high expression in pulmonary epithelium suggests an important role in lung homeostasis^21^. RAGE is also involved in the maintenance of pulmonary structure and alveolar barrier integrity^24^. By analysing the transdifferentiation of AT2 cells into AT1 cells in vitro, Demling et al.^22^ identified RAGE as a specific differentiation marker of human AT1 cells. Although RAGE is expressed in the basolateral membrane of the majority of epithelial cells, interestingly no expression is found in AT2 cells, whereas AT1 cells express high levels of RAGE, thus being a reliable marker for type 1 cells in the alveolar tissue^25^.

Caveolin-1 is the major structural protein within the caveolar vesicles^26^. Caveolin-1 is mainly involved in transcellular (in this case transepithelial) transport via caveolar vesicles which are located on both apical and basal membranes of cells^27^. In the alveolar epithelium, caveolin-1 is highly expressed in AT1 cells^27^. AT2 cells normally express no, or very little, caveolin-1; however, during the transdifferentiation of AT2 cells into AT1 cells, CAV1 expression is highly induced^28^. In the present work, caveolin-1 was expressed in all IC strains and TT1 cells, confirming the AT1 cell phenotype of the immortalised cells. The transcriptional levels of CAV1 in some of the IC preparations differed from that in TT1 cells. Five of the twelve strains showed significantly higher CAV1 transcriptional levels compared to TT1 cells (p < 0.05). Differential expression of CAV1 in ICs may be related to a greater endocytic capacity in those IC strains compared to the TT1 cell line.

Podoplanin was also identified in ICs through immunoblotting and immunocytochemical analysis. According to immunoblots, podoplanin was expressed in eleven of the twelve ICs and also in TT1 cells. Immunocytochemical analysis identified podoplanin positive cells in all ICs and TT1 cells. AT2 cells did not express podoplanin, regardless of method used. Within each HAEL, immunocytochemistry showed podoplanin to be present in most of the IC cells; however, some cells (~ 6%) did not stain for podoplanin, indicating the presence of some AT2-like cells in the IC populations. Podoplanin is a mucin-type transmembrane protein that has a wide variety of functions including organ development, cell motility and proliferation, and is also involved in inflammatory diseases, tumorigenesis and metastasis^29^. It is expressed in several organs such as heart, intestine and lungs^29,30^. In the lung, podoplanin is expressed by AT1, but not by AT2, cells^29^, being involved specifically in the maturation of AT1 cells^30^. Thus, these data show that all the ICs contained a high proportion of cells that expressed podoplanin, an AT1 cell phenotype, reflecting maturation and differentiation of the transformed AT2 cells.

Surfactant protein SP-C, which is expressed by AT2 cells^31^ and not by AT1 cells, was expressed in all the IC lines. Immunoblotting analysis showed that SP-C was present in all ICs and AT2 cells but not in TT1 cells. Gene expression analysis corroborated that TT1 cells did not express SFTPC. All IC strains expressed SFTPC, however five showed statistically higher SFTPC transcription compared to TT1 cells (p < 0.05). As mentioned above, SP-C within the phospholipid-protein complex facilitates lung surfactant absorption at the air–liquid interface, ensuring reduced surface tension during respiration. SP-C is the most exclusive AT2 cell surfactant protein, being synthesised only in AT2 cells, whereas SP-A, B and D are also synthesised by bronchiolar Club cells^31^. In the present study, both gene expression and immunoblotting confirmed the expression of SP-C in ICs. Since IC cells also expressed AT1 cell markers, this suggests that all the ICs contain a proportion of alveolar epithelial cells which exhibit either largely AT1 (a majority of the cells) or largely AT2 phenotypes (a small proportion of the cells), the latter possibly being progenitor cells which can transdifferentiate into AT1-like cells; thus, the evidence herein suggests that all the ICs all contain a proportion of cells which co-express both AT2 and AT1 characteristics, as described to occur in normal lung tissue during the AT2 to AT1 transition^34–38^, and thus that a proportion of the cells in all the ICs are transitional.

Further evidence to support this concept was gained through alkaline phosphatase staining. Alkaline phosphatase is another AT2 cell marker^4,32^ and its expression is regulated in parallel to surfactant proteins^32^. As shown in Fig. 5, primary AT2 cells exhibited strong pink AP-positive staining, unlike TT1 cells, confirming AT1-like phenotype of the latter. Like TT1 cells, ICs were mostly AP-negative; however, some IC strains contained scattered strongly AP-positive cells. Thus, alkaline phosphatase staining also supports the concept that ICs consist of a mixture of cell phenotypes exhibiting varying degrees of AT1-like and AT2-like characteristics.

TEM showed that most ICs exhibited a flattened thin morphology as seen in vivo for AT1 cells. The cytoplasm of these cells was relatively homogeneous, with few cellular organelles, mostly mitochondria and endosomal vesicles within the cells and endosomal invaginations at cell membranes (Fig. 6a–d), all features of AT1 cells in situ. A few cells were more spherical and contained large cytoplasmic vesicles resembling lamellar bodies, indicating that they produce and store surfactant. Lamellar bodies are large organelles unique to AT2 cells that contain the surfactant phospholipids and proteins in a lamellar format ready to release onto the air–liquid interface^5^. These cytoplasmic inclusions are observed in AT2 cells but not in AT1 or in the transdifferentiated TT1 cell line^13^. During transdifferentiation of AT2 cells, the number of lamellar bodies significantly decrease^6^ until they finally disappear on transition to AT1 cells. In the present work, although cells did not show the classic cuboidal shape of AT2 cells, a proportion of cells showed apical microvilli, strong tight junctions and lamellar bodies, typical features of AT2 cells^19,33^, which, alongside the expression of SP-C gene and protein expression, and positive alkaline phosphatase staining, as well as negative podoplanin staining, further supports the presence of a small proportion of largely AT2-like cells within some IC populations which may, nevertheless, differentiate into AT1-like cells. Other cells within the ICs contained structures resembling lamellar bodies, but were not cuboidal, they were flattened like AT1 cells, thus showing both AT1 and AT2 characteristics, representing a transient phenotype between AT2 and mature AT1-like cells (Supplementary Fig. S3). This aligns with recent studies in mice which indicate that a subpopulation of alveolar epithelial cells, expressing markers of both AT1 and AT2 cells, exists in the alveolar epithelium^34–37^, which can differentiate into mature AT1 or AT2 cells, upregulating markers of the corresponding cell fate and downregulating markers of the alternative cell fate^34–37,39^. These progenitor cells may well exist in some of these IC lines. This needs further exploration.

Although there are a number of studies describing subpopulations of alveolar epithelial stem cells and mechanisms of differentiation^34–37,39^, the majority have been performed in mice, particularly during postnatal alveolarisation (which in humans occurs in the foetus). In humans it is unclear whether equivalent subpopulations of stem/progenitor cells exist and which mechanisms control cell differentiation and lung tissue regeneration in the adult lung. In the ICs, the continued presence at each passage of a number of cells with AT2 characteristics suggests that these may correspond to such progenitor cells.

Further, the proliferation rate was assessed for ICs maintained over 15 days in culture. TT1 cells were grown under the same conditions for comparative purposes. ICs showed a faster rate of propagation compared to TT1 cells, especially during the first 24 h. ICs achieved monolayer confluency after approximately 5 days in culture and remained confluent beyond fifteen days. TT1 cells achieved monolayer confluency later (6 days) than some ICs but also remained confluent beyond fifteen days. Witherden and Tetley^4^ reported that primary human AT2 cells achieve monolayer confluency after 2 to 3 days in culture; however in the previous work, cells were seeded at much higher density than in the present work because primary AT2 cells do not proliferate adequately when thinly seeded. Similarly, A549 cells seeded at much higher cell density, achieved monolayer confluency after 7 to 10 days in culture^40,41^. Thus, the ICs replicate more rapidly than other alveolar cells in vitro, and are good candidates for short and medium-term in vitro studies.

To validate ICs as cellular models for toxicity screening, cells were exposed to positively charged PS-NH_2_ and negatively charged PS-COOH particles of the same size (50 nm). TT1 cells were also exposed to the same particles for comparative purposes. ICs were highly sensitive to PS-NH_2_ at all concentrations tested whereas PS-COOH particles were not cytotoxic at any of the concentrations tested. As previously reported, PS-NH_2_ are particularly cytotoxic for several mammalian cell lines^40–46^ including the human alveolar TT1 cells^47,48^ and A549 cells^49^. As shown by Anguissola et al.^50^, toxicity of PS-NH_2_ particles is mainly driven by their surface charge, causing lysosomal destabilisation, loss of mitochondrial potential and decrease in plasma membrane integrity. This toxicity can be tempered by suspending PS-NH_2_ in a protein rich medium as adsorption of proteins reduces their surface charge and toxicity^46,50,51^. The findings in the present work for TT1 cells are consistent with previous work published by us^47^. Comparing ICs with other cell lines, ICs were more sensitive to low PS-NH_2_ concentrations than TT1 cells (Fig. 8; Ruenraroengsak and Tetley^47^) and were generally more sensitive than A549 cells at all PS-NH_2_ doses^49^; thus the newly generated ICs represent valuable, sensitive cell models for in vitro toxicity testing.

Finally, comparing cell lines generated using viral vector (TT1 cells) with ICs generated with non-viral methods (HAEL), the latter have proved to better mimic the alveolar epithelium of lung tissue. While TT1 cells are composed exclusively of cells expressing AT1 cell characteristics, HAEL have shown cells expressing AT1 (majority of cells) and AT2 cell characteristics, thus maintaining the cellular heterogeneity that naturally occurs in the alveolar epithelium. Additionally, HAEL have been shown to conserve strong tight junctions between cells, a particular feature of AT2 cells, which makes them better candidates for studies involving e.g. particle translocation across gas-blood alveolar barrier and barrier integrity.

Viral vectors generally show higher transfection efficiency compared to non-viral methods. However, the main drawbacks of using viral vectors are their immunogenicity, cytotoxicity and their potential risk to cause insertional mutagenesis leading to the malignant transformation of cells. This paper describes an optimised non-viral method that guaranties high transfection efficiency for the generation of immortalised alveolar epithelial lung cells alongside reduced pathogenicity, low cost and increased biosafety by avoiding the use of viral vectors.

In conclusion, we have developed a new technique to immortalise primary AT2 cells using a non-viral vector. This technique showed high efficiency in gene delivery, improving safety over the use of viral vectors, thus representing a cost-effective alternative. As summarised in Table 2, newly immortalised cells showed mostly AT1 cell characteristics such as expression of RAGE, caveolin-1 and podoplanin and alkaline phosphatase negativity. Thus, a significant degree of AT2 transdifferentiation occurred as a result of the immortalisation procedure. Nevertheless, scattered AP-positive cells were present and TEM highlighted cells exhibiting cytoplasmic lamellar bodies, apical microvilli and tight junctions were observed, indicative of the presence of cells expressing AT2 cell characteristics. The strong gene expression of the AT2 cell marker SP-C by some of the cell lines indicates the existence of AT2 transitional cells in some of these cell lines possibly representing progenitor cells expressing a strong AT2 phenotype that may have the capacity to transdifferentiate into AT1-like cells (i.e.the majority of cells in the IC strains). This heterogeneity is not a consequence of the method used to transform the cells, but an inherent property of these cells which occurs in normal alveolar lung tissue during the transdifferentiation of AT2 cells into AT1 cells. Finally, ICs proved an effective in vitro model to investigate the potential effects of toxicants. The differential expression between the HAELs of specific cellular processes and characteristics also offers exciting opportunities for comparative studies of specific cell behaviour, not yet investigated. Thus, this novel approach for immortalisation of primary human alveolar cells has generated a series of cell lines that exhibit important features that mimic the human alveolar epithelium and can be used for toxicological and, we suggest, pharmaceutical purposes. In addition, these models offer exciting opportunities to study the behaviour of human alveolar epithelium in vitro, under normal and pathogenic conditions, which is currently significantly limited by the availability of human material.

**Table 2:** Summary of characteristic features positively expressed (+), negatively expressed (−) and negative with scattered positive expression (−/S +) in ICs compared with primary AT2 cells and the type 1 cell line (TT1).

Techniques	AT2	TT1	HAEL-1	HAEL-2	HAEL-3	HAEL-4	HAEL-5	HAEL-6	HAEL-7	HAEL-8	HAEL-9	HAEL-10	HAEL-11	HAEL-12
**Gene expression**														
RAGE	−	+	+	+	+	+	+	+	+	+	+	+	+	** + **
Caveolin-1	−	+	+	+	+	+	+	+	+	+	+	+	+	** + **
SP-C	+	−	+	+	+	+	+	+	+	+	+	+	+	** + **
**WBs**														
RAGE	−	+	+	+	+	+	+	+	+	+	+	+	+	** + **
Caveolin-1	−	+	+	+	+	+	+	+	+	+	+	+	+	** + **
Podoplanin	−	+	+	+	−	+	+	+	+	−	+	+	+	** + **
SP-C	+	−	+	+	+	+	+	+	+	+	+	+	+	** + **
**ICC**														
Podoplanin	−	+	+	+	+	+	+	+	+	+	+	+	+	** + **
Alkaline phosphatase activity	+	−	−/S +	−/S +	−	−	−/S +	−/S +	−/S +	−/S +	−/S +	−/S +	−/S +	−
**TEM**														
Membrane invaginations	-	+	+	+	+	+	+	+	+	+	+	+	+	** + **
Apical microvilli	+	−	−/S +	−/S +	−	−/S +	−	−	−	−	−	−	−/S +	−/S +
Tight junctions	+	−	−	−	−	−/S +	−	−	−	−	−	−	−/S +	−/S +
Lamellar Bodies	+	−	−/S +	−/S +	−	−/S +	−	−	−	−	−/S +	−/S +	−/S +	−/S +

Gene expression data and data on the ultrastructure of AT2 cells were obtained respectively from Johansson et al.^28^and Bingle et al.^6^.

## Methods

### Isolation of primary AT2 cells

Lung tissue was obtained from one adult donor in the Royal Brompton Hospital (London, UK) with informed, signed consent of the patient. All methods were carried out in accordance with relevant guidelines and regulations. Ethical approval was given by NRES Committee South Central—Hampshire B REC reference: 15/SC/0101. Primary AT2 cells were isolated according to Witherden and Tetley^4^ and Witherden et al.^19^.

### Transfection of primary AT2 cells

Freshly isolated primary human AT2 cells were seeded to be 30%, 50% and 70% confluent in a 24-well microplate previously coated with 1% PureCol collagen solution (Advanced Biomatrix, San Diego, USA). Cells were kept in complete DCCM-1 medium (10% NCS) for 24 h at 37 °C to establish the primary cell culture. After that, medium was removed and cells were gently washed with PBS before the transfection of the cells. For the transfections, lipofectamine 3000 (LF) was diluted in Opti-MEM Medium. Plasmid DNA sequences hTERT (catalytic subunit of telomerase) and SV40 (temperature-sensitive mutant of simian virus 40 large-tumour antigen) were diluted in Opti-MEM medium, mixed with P3000 Reagent and added to the LF dilutions at the ratios: 1:1, 1:2 and 1:4 (LF:DNA). Mixtures were then incubated for 5 min in order to allow LF particles to uptake plasmid DNA before being added to the primary cells. Cells were incubated for 2 days with the mixtures. Then, medium was replaced with complete DCCM-1 supplemented with 0.5 mg/mL G418 (Sigma, Poole, UK) for selection of transfected cells. After 5 days, successfully transfected cells were passaged to a new 1% PureCol coated 24-well microplate and left until cells reached confluency.

### Characterisation of the transfection vector

#### Dynamic light scattering (DLS)

Hydrodynamic diameter and zeta potential were assessed on different dilutions of LF in Opti-MEM medium (1:33, 1:16 and 1:8) and suspensions of LF mixed with plasmid DNA constructs hTERT and SV40 at the ratios: 1:1, 1:2 and 1:4. DLS analysis was performed using a Zetasizer Nano (Malvern Instruments Ltd, UK). The data are expressed as mean ± SD.

#### Transmission electron microscopy (TEM)

LF particles were mixed with the plasmid DNA constructs hTERT and SV40, incubated for 5 min. The samples were placed onto TEM grid and stained with 1% uranyl acetate and the excess sample was adsorb with filter paper before being analysed under a JEOL 2000FX transmission electron microscope operated at 80 kV. The photomicrographs were taken at magnification ranging between × 20,000 to × 100,000.

### Characterisation of the immortalised cells (ICs)

#### Gene transcription level analysis

Due to the low availability of lung tissue to obtain primary AT2 cells, gene transcription analysis was performed only in the cell lines. HAEL and TT1^13^ were grown to confluence on 6 well Nunc microplates (Costar, Corning Life Sciences, Schipol RDK, The Netherlands). Total RNA was extracted from the ICs and TT1 cell line using TRI Reagent solution (Ambion, Life Technologies) following manufacturer’s instructions. cDNA synthesis was performed using the Affinity Script Multiple Temperature cDNA Synthesis Kit (Agilent Technologies) according to manufacturer’s instructions. Quantitative real-time PCR (qPCR) was performed in a 7300 Real-Time PCR thermocycler (Life Technologies) using SYBR Green master mix (Roche Diagnostics, Indianapolis, USA) and predesigned primers available in GenBank database for the target genes RAGE, caveolin-1 and SP-C (Supplementary Table S1 online). Transcriptional levels of the target genes were quantified using the comparative threshold cycle (CT) method^52^. Results were normalised with the amount of cDNA (in ng) charged in the qPCR according to Rojo-Bartolomé et al.^52^.

### Western blotting

ICs, TT1 cell line and primary AT2 cells seeded in 6 well plates were lysed with CellLytic reagent (Sigma-Aldrich, Poole, UK) containing a protease inhibitor cocktail P8340 (Sigma-Aldrich, Poole, UK**)**. Protein content was quantified using NanoDrop platform spectrophotometer **(**Thermo Scientific, Waltham, UK**)** and adjusted to 50 µg per sample. Proteins were resolved on 10**%** NuPAGE Bis–Tris gels **(**Invitrogen, Paisley, UK**)** and transferred to nitrocellulose membranes using an iBlot 7-min Blotting System (Invitrogen, Paisley, UK). Membranes were blocked for 1 h with 5% bovine serum albumin (BSA) (Sigma-Aldrich, Poole, UK) in TBST buffer (20 mM Tris; 50 mM NaCl; 0.1% Tween-20), washed with TBST trice and incubated with 5% BSA in TBST probed with the primary antibody (Supplementary Table S2 online) at 4 °C overnight**.** Then, membranes were washed with TBST three times and incubated with 5% BSA in TBST probed with the secondary antibody (Supplementary Table S2 online) for 1 h at room temperature and washed as before. Finally, membranes were exposed to an enhanced chemiluminescence reagent (ECL) Prime (GE Healthcare, Little Chalfont, UK) for 1 min and developed on X-ray films.

### Immunocytochemistry

Immunocytochemical staining for podoplanin was performed in ICs and TT1 cell line and in primary AT2 cells. Cells were grown to confluence on an 8 well Nunc Lab-Tek II Chamber Slide system (Thermo Fisher Scientific, East Grinstead, UK) previously treated with 1% PureCol collagen solution (Advanced Biomatrix, San Diego, USA). Cells were rinsed with PBS, fixed with 4% paraformaldehyde in PBS for 10 min, washed trice with PBS and permeabilised with 0.2% Triton-X in PBS for 50 min. A blocking step of 1 h in 1% BSA in PBS was performed before the incubation with the antibodies. Monolayers were incubated overnight at 4 °C with a primary mouse monoclonal antibody to podoplanin (ab10288, Abcam, Cambridge, UK) diluted in PBS containing 1% BSA (1:500). Cells were then washed three times with PBS and incubated with a fluorochrome coupled secondary antibody Alexa Fluor 488 goat anti-mouse (ab150113, Abcam, Cambridge, UK) diluted in PBS containing 1% BSA (1:500) for 1 h. Unbound secondary antibody was washed with PBS and cells nuclei stained with 1 μg/mL Hoescht (Sigma Aldrich, Poole, UK). Confocal images were obtained using a Leica SP5 inverted confocal microscope.

### Alkaline phosphatase (AP) staining

Alkaline phosphatase staining was previously described as a positive marker for AT2 cells phenotype^4,19^. The AP staining was carried out on ICs and TT1 cell line and primary AT2 cells according to Witherden and Tetley^4^. Cells were analysed following inverted confocal microscopy as described above.

### Transmission electron microscopy (TEM)

Cells were grown to confluence on clear polyester Transwell membranes (0.4 mm pore size; Corning Life Sciences, Schipol RDK, The Netherlands) and prepared for TEM analysis following the protocol described in Ruenraroengsak and Tetley^47^. Sections were observed in a JEOL JEM-1230 transmission electron microscope operated at 80 kV. The photomicrographs were taken at magnification ranging between 10,000 × to 100,000x.

### Cell proliferation

Cell proliferation was measured using electrical cell-substrate impedance sensing (ECIS) in an iCELLigence System (ACEA Biosciences Inc., San Diego, USA). The presence of cells on top of the ECIS electrodes affects the ionic environment between the electrode and the solution, leading to an electrode impedance. The increase in the number of cells on the electrodes leads to an increase in electrode impedance. Electrode impedance is displayed as Cell Index values and correlates with the extent of cell numbers attached to the bottom surface. ICs and TT1 cell line were seeded at 100 cells/well in duplicate and cultured in complete medium. Culture medium was replaced every three days and cell proliferation was monitored over 15 days.

### Exposure of ICs and TT1 cell lines to polystyrene nanospheres and assessment of cell viability

ICs and TT1 cell line were grown to confluence in 96-well microplates and then serum starved 24 h before the exposures. Cells were exposed for 24 h to 25, 50, 75 and 100 μg/mL of 50 nm polystyrene nanospheres (Sigma Aldrich, Poole, UK) either positively charged (amine modified, PS-NH_2_) or negatively charged (carboxylate modified, PS-COOH) suspended in serum-free medium. Unexposed cells were used as control. Following the exposures, medium was removed and cells washed with PBS to remove residual nanospheres and cell viability was assessed using the MTT assay (mitochondrial activity; Thermo Fisher Scientific, East Grinstead, UK) according to manufacturer’s instructions. Absorbance was measured at 570 nm in a SPECTRAFluor Plus microplate spectrophotometer (TECAN, Milan, Italy). Six replicates per exposure condition were used and experiments were repeated three times each.

### Statistics

Differences in the transcriptional levels of selected genes in ICs and TT1 cell lines and differences between control cells and cells exposed to polystyrene nanospheres were assessed through the Kruskal–Wallis test followed by the Dunn's post hoc test using the R 3.5.0 statistical software (freely available at https://www.r-project.org/). Significance level was globally established at 5% (p < 0.05). LC_50_ values obtained in the cell viability assay were calculated through Probit analysis using the SPSS 23.0 software (Chicago, USA; licenced through Imperial College London, ICT centre).

## Supplementary information

Supplementary Information.

## Data Availability

The source data underlying Figures, Tables and Supplementary Information are available from the authors upon request.
