# Supplementary material for: Immortalisation of primary human alveolar epithelial lung cells using a non-viral vector to study respiratory bioreactivity in vitro
Source: Sci Rep. 2020 Nov 24;10:20486. doi: 10.1038/s41598-020-77191-y (PMC7686381; doi:10.1038/s41598-020-77191-y)
Supplement: Supplementary file 1 — Supplementary Information. [file 41598_2020_77191_MOESM1_ESM.pdf]

Immortalisation of primary human alveolar epithelial lung cells using a non-viral vector to study respiratory bioreactivity *in vitro*

Alberto Katsumiti[1, 2*], Pakatip Ruenraroengsak[3, 4], Miren P. Cajaraville[1], Andrew J. Thorley[2, 5], Teresa D. Tetley[2*]

Supplementary Information

Figure S1. Hydrodynamic size measured by DLS of LF in Opti-MEM medium at 1:33, 1:16 and 1:8 (LF: Opti-MEM) (A-C) and of LF-DNA nanomeric complexes at 1:1, 1:2 and 1:4 (LF:DNA) dilution ratios. n = 3 replicates per sample.

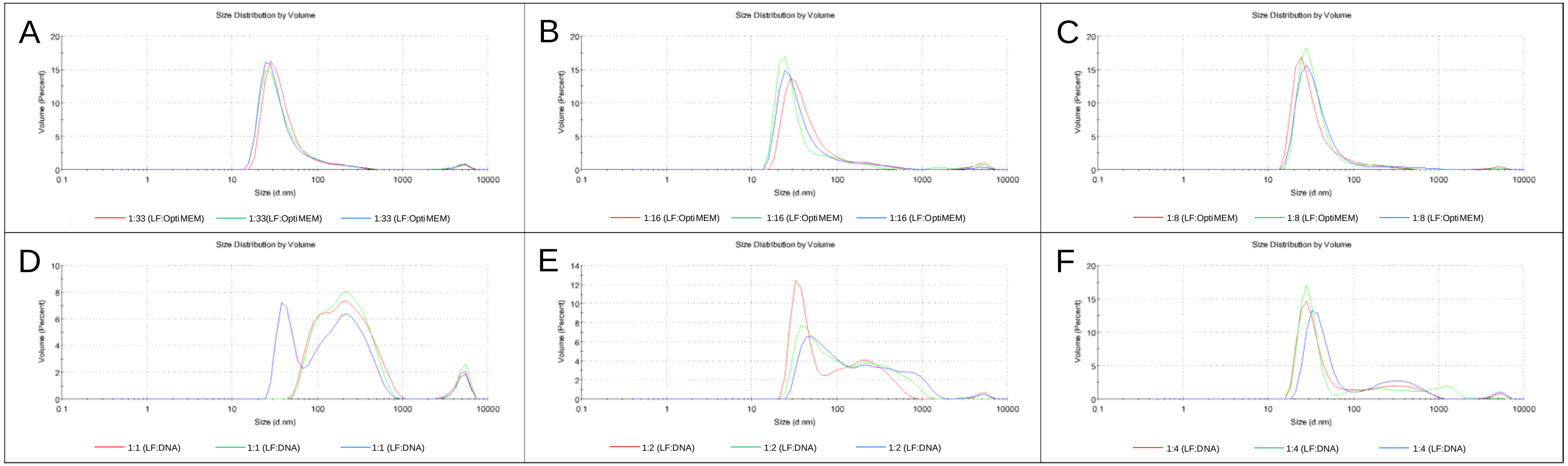

Figure S2. Full-length original and unprocessed immunoblots of RAGE, caveolin-1, podoplanin and SP-C in ICs, TT1 cell line and primary AT2 cells. Loading protein concentrations were adjusted to 50 µg per sample.

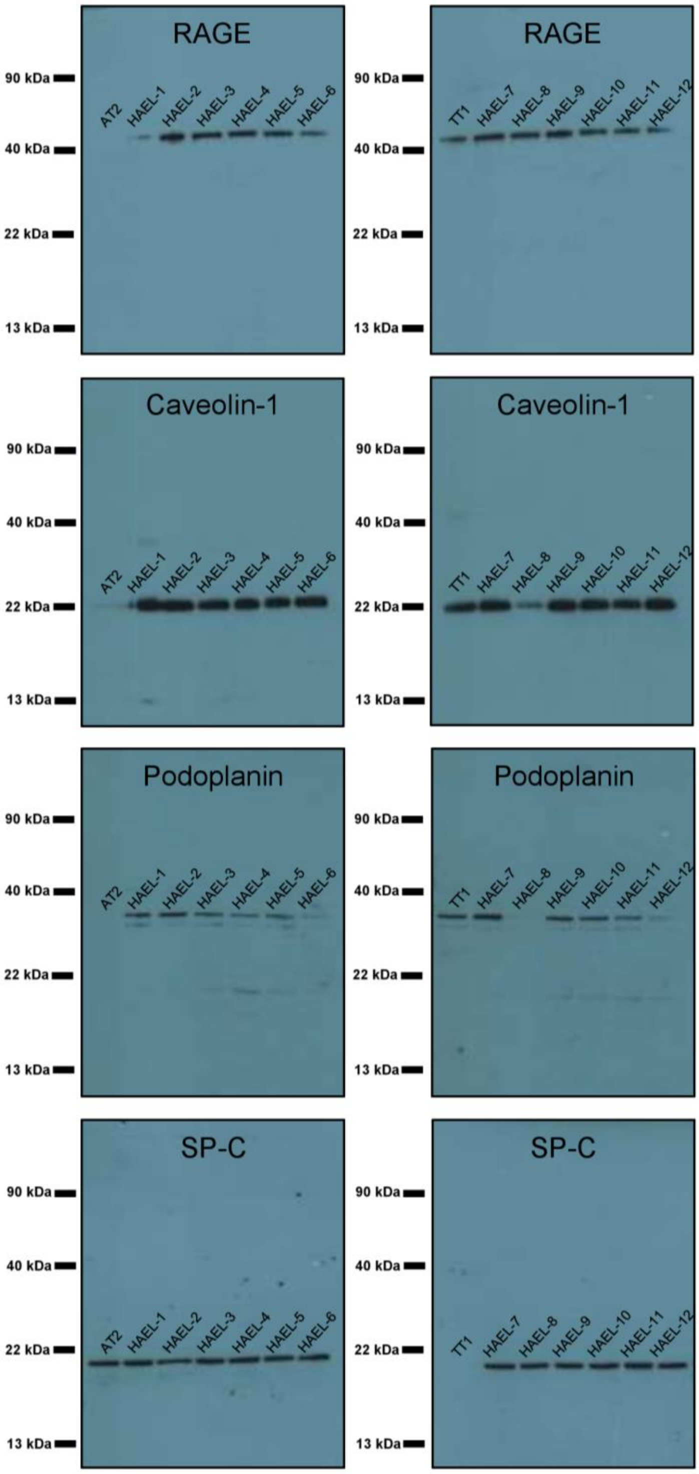

Figure S3. A cell showing structures resembling lamellar bodies (*) but flattened AT1 morphology, thus showing both AT1 and AT2 characteristics,

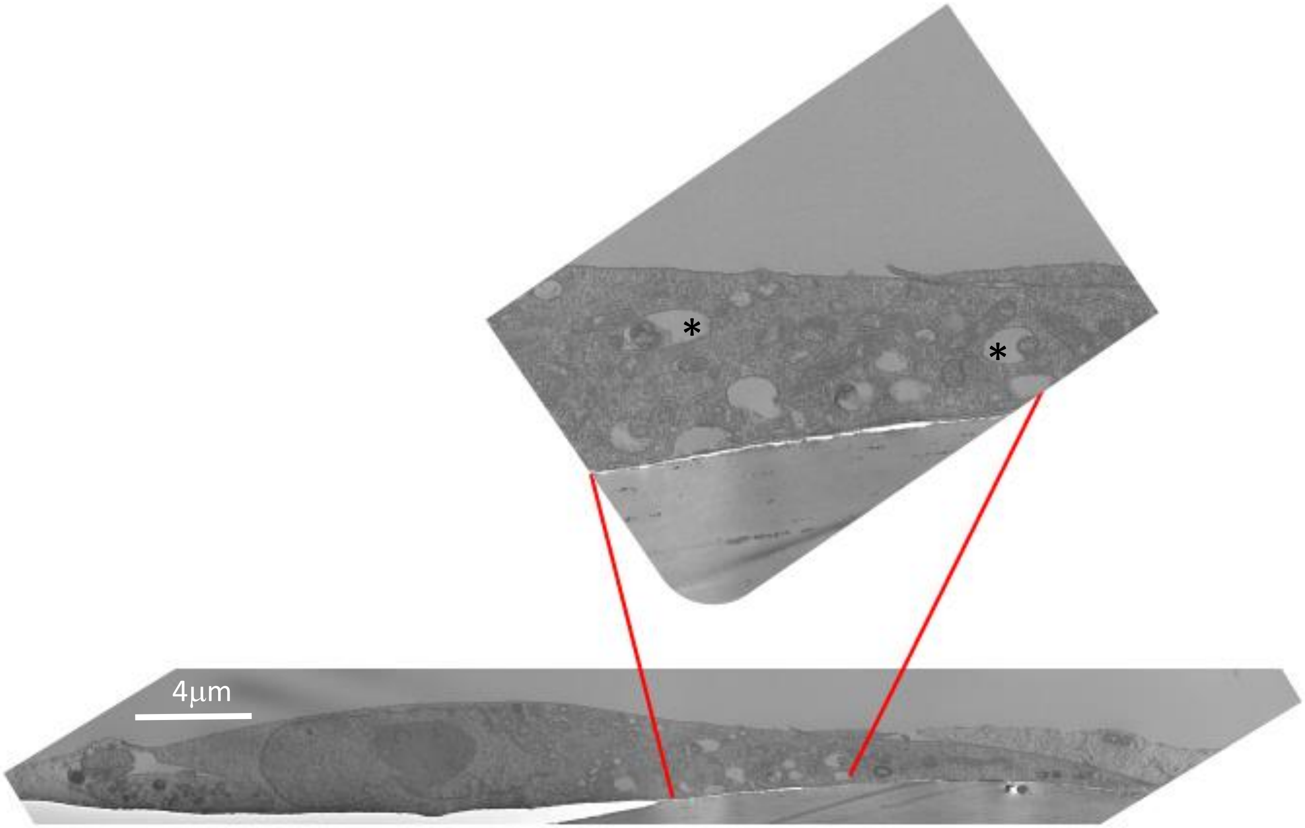

Table S1. Target genes and primer sequences used for the qPCR analysis and their respective functions in the lung tissue.

Target gene	Gene name	Gene ID	Accession number	Forward primer (5’-3’)	Reverse primer (3’-5’)	Function
RAGE	*AGER*	177	NM_001136.4	CTACCGAGTCCGTGTCTACC	TTCTCATTAGGCACCAGGGG	Inflammatory response
Caveolin-1	*CAV1*	857	NM_001753.4	CGTAGACTCGGAGGGACAT	GTTGAGGTGTTTAGGGTCGC	Transepithelial transport
SP-C	*SFTPC*	6440	NM_003018.3	CCTGAGTGAGCACCTGGTTA	TCAAGACTGGGGATGCTCTC	Maintenance of alveolar homeostasis

Table S2. Primary and secondary antibodies and their respective dilutions used for Western blot analysis.

	**Primary antibody**	**dilution factor**	**Secondary antibody**	**dilution factor**
**RAGE**	Rabbit polyclonal anti-human receptor for advanced glycation endproducts (Abcam, Cambridge, UK)	1:10,000	Polyclonal goat anti-rabbit (Dako, Ely, UK)	1:25,000
**Caveolin-1**	Rabbit polyclonal anti-human caveolin-1 (Abcam, Cambridge, UK)	1:1,000	Polyclonal goat anti-rabbit (Dako, Ely, UK)	1:25,000
**Podoplanin**	Mouse monoclonal anti-human podoplanin (Abcam, Cambridge, UK)	1:10,000	Polyclonal goat anti-mouse (Dako, Ely, UK)	1:25,000
**SP-C**	Rabbit polyclonal anti-human prosurfactant protein C (Abcam, Cambridge, UK)	1:10,000	Polyclonal goat anti-rabbit (Dako, Ely, UK)	1:25,000
